# Diagnosis and surgical treatment of isolated rectal endometriosis: long term complication of incomplete treatment for pelvic endometriosis

**DOI:** 10.12669/pjms.323.9492

**Published:** 2016

**Authors:** Jae-Young Kwack, Seul Ki You, Yong-Soon Kwon

**Affiliations:** 1 Jae-Young Kwack, Departments of Obstetrics and Gynecology, University of Ulsan College of Medicine, Ulsan University Hospital, Ulsan, Korea; 2 Seul Ki You, Departments of Obstetrics and Gynecology, University of Ulsan College of Medicine, Ulsan University Hospital, Ulsan, Korea; 3 Yong-Soon Kwon, Departments of Obstetrics and Gynecology, University of Ulsan College of Medicine, Ulsan University Hospital, Ulsan, Korea

**Keywords:** Cyclic hematochezia, Pelvic endometriosis, Rectal endometriosis

## Abstract

A 40-year-old woman visited our hospital with cyclic hematochezia for four months. The patient had the history of laparoscopic-assisted vaginal hysterectomy because of severe dysmenorrhea two years ago at another tertiary hospital. According to the medical records, the past surgical treatment was incomplete excision of pelvic endometriotic lesions, especially in rectal serosal lesions.

A colonoscopy and abdominopelvic computed tomography showed an isolated tumor mimicking neoplasm, in which a biopsy under colonoscopy was performed and the lesion was endometriosis pathologically. Laparoscopic anterior resection (LAR) was performed. There were no complications during intraoperative and postoperative period and the patient was discharged 7 days after the LAR. It is important for reducing of long-term complication like rectal endometriosis that complete and safe excision of pelvic endometriosis with expert surgical strategy.

## INTRODUCTION

Endometriosis is a common disease in women of reproductive age and the severity of the disease is diverse. Most of severe pelvic endometriosis involved cul-de-sac, serosa of rectum, and posterior surface of uterus. Intestinal endometriosis is rare and it is more frequently located in the rectosigmiod (50-90%).^1^,^2^ Sometimes, it is difficult to accomplish a complete surgical excision of severe pelvic endometriosis, especially hard, firmly adhesion between uterus and rectum via laparoscopy or laparotomy. We report an isolated rectal endometriosis after surgical hysterectomy with cyclic hematochezia because of an incomplete excision of pelvic endometriosis.

## CASE REPORT

A 40-year-old woman presented consecutively cyclic hematochezia for four months on visiting our hospital. The cyclic hematochezia was associated with low abdominal discomfort and tenesmus. The past surgical history of the patient was a laparoscopic-assisted vaginal hysterectomy with right salpingo-oophorectomy for severe pelvic endometriosis with severe dysmenorrhea two years ago at another tertiary hospital. According to the medical records, there was an injury of right ureter during the operation, which was corrected with end to end anastomosis via a laparotomic conversion and incomplete excision of pelvic endometriotic lesions, especially in rectal serosal lesions. The patient had received 6 cycles GnRH agonist postoperatively.

**Fig.1:**
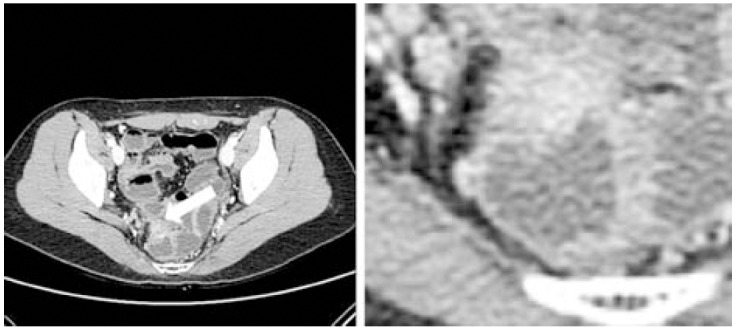
AP-CT showed a 1.5-cm sized mural nodule with heterogeneous hyperattenuation, located at mid rectum right-anterior wall; The mass was a lobulated exophytic contour with eroded mucosa & mild perirectal fat invasion. B: Magnificating view of rectal endometriosis lesion.

**Fig.2:**
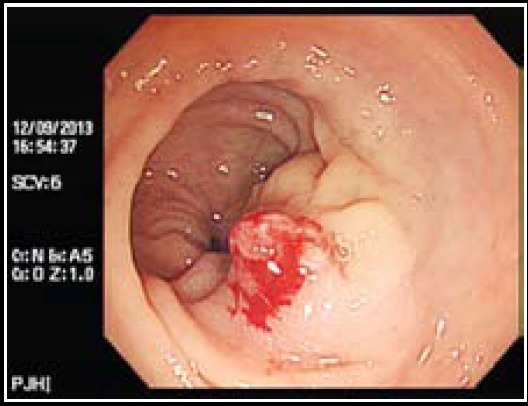
Colonoscopic view: the isolated, tranmural endometriotic lesion at anoverge 7 cm.

A colonoscopy was performed for detecting the cause of hematochezia and showed submucosal mass mimicking neoplasm with bleeding at 7 cm above the anal verge (Fig.1). Under colonoscopy, a biopsy was performed and pathologic report confirmed rectal endometriosis. AP-CT was taken to detect another lesions, and it showed an isolated tumor mimicking neoplasm on the same area of rectum without other site-lesions (Fig.2).

**Fig.3:**
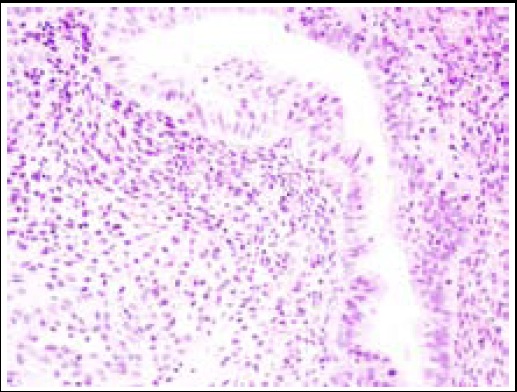
Rectal mass obtained by colonoscopic biopsy, which showed stroma and glands of endometriosis (hematoxylin and eosin, 400x).

Laparoscopic anterior resection (LAR) was performed by an experienced colorectal surgeon with thorough inspection of other site lesions into pelvic cavity. Finally, permanent pathologic report revealed rectal endometriosis with clear resection margins (Fig.3). There were no complications during intraoperative and postoperative period and the patient was discharged seven days after the LAR. The patient received 6 cycles GnRH agonist after the LAR and were followed up for 8 months with clinical no evidence of disease proven by colonoscopy and AP-CT.

**Fig.4:**
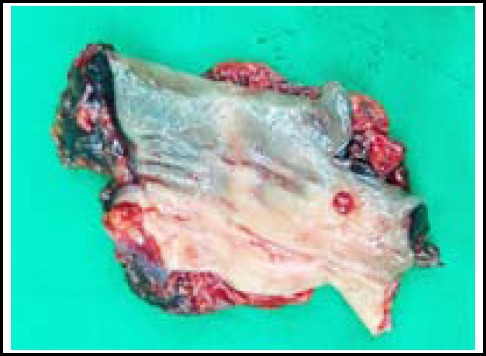
The obtained specimen after laparoscopic anterior resection of the endometriosis involved rectum: the arrow indicated the isolated rectal endometriotic lesion.

**Fig.5:**
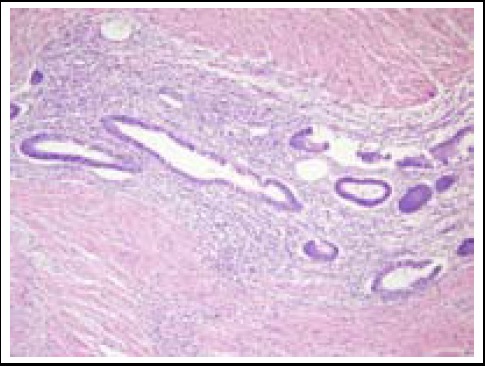
Endometriosis composed of stroma and glands of endometriosis into rectal wall (hematoxylin & eosin, 100x).

## DISCUSSION

Intestinal endometriosis is rare but is possible on every layer of the bowel, which is almost superficial infiltration as subserosal implants.^3^,^4^ Transmural involvement of endometriosis is uncommon.^2^ In the current case, the hysterectomized woman with incomplete excision of pelvic deep infiltrating endometriosis into rectum and the lesion develops due to hormonal activity from preserved left ovary because of ineffective postoperative hormonal treatment.

Clinical presentations of rectal endometriosis are dependent on the severity and extent of endometriosis on the involved rectum. These clinical symptom could be asymptomatic or it might present with diffuse abdominal pain, bloating, constipation, diarrhea, change in the form and caliber of stool, tenesmus, and intestinal obstruction.^1^,^5^-^7^ A clue symptom of transmural rectal endometriosis is menstruation-like cyclic hematochezia, tenesmus, and diarrhea with regular monthly interval. In the current case, the patient had abrupt onset of cycle hematochezia with the disease advancement.

Except cyclic symptoms of transmural deep infiltrating endometriosis, the diagnosis of intestinal endometriosis is greatly challengeable because of non-specific symptoms. Frequently, diagnosis of non-specific intestinal endometriosis can be possible by screening AP-CT for personal health-care program. In patients with symptomatic deep infiltrating endometriosis, a primary tool for diagnosis is colonoscopy, which can obtain direct visual evidence of lesion and subsequently direct biopsy. However, it could be confused with malignancy, based on the results of colonoscopy and radiologic scan, particularly in patients with mucosal involvement.^5^-^8^ Therefore, it is very important for accurate diagnosis to perform a direct biopsy under colonoscopic examination. The pathologic diagnosis of transmural intestinal endometriosis by colonoscopic biopsy was done in our case.

The therapeutic strategy for intestinal endometriosis is not yet established and is dependent on the extent and symptoms of the disease. However, surgical treatment should be considered in the cases, which included the deep infiltrating intestinal endometriosis with recurrent or disabling symptoms to differentiate between endometriosis and neoplasm of the bowel, and to eliminate the lesion of bowel endometriosis invaded more than 50% of the bowel circumference or the bowel depth.^3^,^5^,^9^

## CONCLUSION

Deep infiltrating endometriosis has frequently been managed by laparoscopic approach, like laparoscopic hysterectomy with/without salpingo-oophorectomy, but complete excision of deep infiltration endometriosis should not always be carried out. Most of these cases are women with reproductive age, who have premenopausal hormonal status affecting on the remnant endometriotic lesions. Despite of previous hysterectomy with/whithout salpingoophorectomy in woman with deep infiltrating endometriosis, it is important to perform regular follow-up. Clinicians have to pay attention to the symptoms that can be caused by remnant lesions or by relapsed pelvic endometriosis such as bowel endometriosis. Also, multidisciplinary approach involving colorectal surgeon is needed for proper management of the patients.
